# NEW MEASURES AND POPULATION PREVALENCE OF VISION IMPAIRMENT IN NHATS

**DOI:** 10.1093/geroni/igad104.0764

**Published:** 2023-12-21

**Authors:** Niranjani Nagarajan, Joshua Ehrlich

**Affiliations:** University of Michigan, Ann Arbor, Michigan, United States; University of Michigan, Ann Arbor, Michigan, United States

## Abstract

The National Health and Aging Trends Study (NHATS) is a nationally representative panel study of older U.S. adults that in 2021 (Round 11) added to its annual protocol a suite of objective visual function measures consisting of distance visual acuity, near visual acuity, and contrast sensitivity tests. These measures were developed to approximate clinical gold-standard tests, while allowing for administration on a tablet in participants’ homes. The tests were found to have good agreement with clinical gold-standards based on their limits of agreement, correlations, and lack of systematic bias between tests. In the initial 2021 fielding of the NHATS visual function measures, 27.8% (25.5%-30.1%) of U.S. adults over age 70 had one or more visual impairment (VI) (distance VI: 10.3% [8.9%-11.7%]; near VI: 22.3% [20.3%-24.3%]; contrast sensitivity impairment: 10.0% [8.5%-11.4%]), and the prevalence was highest among those who were older, had less education, lower income, and were of Hispanic ethnicity and non-White race. Additionally, the adjusted prevalence ratio for dementia was significantly higher among those with each type of VI compared to those with normal vision (moderate/severe distance VI=1.72 [1.26-2.35]; any near VI=1.40 [1.16-1.69]-; contrast sensitivity impairment =1.31 [1.04-1.66]). Since few other national studies of older adults include objective visual function measures, NHATS now serves as a vital resource for the study of vision epidemiology and the impact of VI and blindness on later-life health outcomes and trajectories.

